# Adaptive magnetic resonance image guided radiation for intact localized prostate cancer how to optimally test a rapidly emerging technology

**DOI:** 10.3389/fonc.2022.962897

**Published:** 2022-09-05

**Authors:** William A. Hall, Amar U. Kishan, Emma Hall, Himanshu Nagar, Danny Vesprini, Eric Paulson, Uulke A. Van der Heide, Colleen A. F. Lawton, Linda G. W. Kerkmeijer, Alison C. Tree

**Affiliations:** ^1^ Department of Radiation Oncology, Medical College of Wisconsin, Milwaukee, WI, United States; ^2^ Department of Radiation Oncology, University of California, Los Angeles, Los Angeles, CA, United States; ^3^ Division of Radiotherapy and Imaging, The Institute of Cancer Research, London, United Kingdom; ^4^ Depart of Radiation Oncology, Weill Cornell Medicine, Department of Radiation Oncology, New York, NY, United States; ^5^ Department of Radiation Oncology, Sunnybrook Hospital, University of Toronto, Toronto, ON, Canada; ^6^ Department of Radiation Oncology, The Netherlands Cancer Institute, Amsterdam, Netherlands; ^7^ Department of Radiation Oncology, Radboud University Medical Center, Nijmegen, Netherlands; ^8^ The Royal Marsden NHS Foundation Trust, and the Institute of Cancer Research, Sutton, United Kingdom

**Keywords:** MR guided radiation therapy, prostate cancer, adaptive radiation therapy prostate cancer, adaptive radiation therapy, FLAME prostate, MR guided radiation prostate cancer, MIRAGE trial

## Abstract

**Introduction:**

Prostate cancer is a common malignancy for which radiation therapy (RT) provides an excellent management option with high rates of control and low toxicity. Historically RT has been given with CT based image guidance. Recently, magnetic resonance (MR) imaging capabilities have been successfully integrated with RT delivery platforms, presenting an appealing, yet complex, expensive, and time-consuming method of adapting and guiding RT. The precise benefits of MR guidance for localized prostate cancer are unclear. We sought to summarize optimal strategies to test the benefits of MR guidance specifically in localized prostate cancer.

**Methods:**

A group of radiation oncologists, physicists, and statisticians were identified to collectively address this topic. Participants had a history of treating prostate cancer patients with the two commercially available MRI-guided RT devices. Participants also had a clinical focus on randomized trials in localized prostate cancer. The goal was to review both ongoing trials and present a conceptual focus on MRI-guided RT specifically in the definitive treatment of prostate cancer, along with developing and proposing novel trials for future consideration. Trial hypotheses, endpoints, and areas for improvement in localized prostate cancer that specifically leverage MR guided technology are presented.

**Results:**

Multiple prospective trials were found that explored the potential of adaptive MRI-guided radiotherapy in the definitive treatment of prostate cancer. Different primary areas of improvement that MR guidance may offer in prostate cancer were summarized. Eight clinical trial design strategies are presented that summarize options for clinical trials testing the potential benefits of MRI-guided RT.

**Conclusions:**

The number and scope of trials evaluating MRI-guided RT for localized prostate cancer is limited. Yet multiple promising opportunities to test this technology and potentially improve outcomes for men with prostate cancer undergoing definitive RT exist. Attention, in the form of multi-institutional randomized trials, is needed.

## Introduction

Localized prostate cancer is increasing in incidence and represents a major oncologic burden worldwide (1). Fortunately, there are several highly effective therapeutic strategies for men with localized prostate cancer (2, 3). In most cases, localized prostate cancer is highly curable with minimal morbidity. Radiation therapy (RT) represents an effective and curative modality for men with prostate cancer, ranging from low to very high risk, for which the outcomes are excellent. As an example, in men with high risk prostate cancer, rates of five year biochemical control with RT and androgen deprivation therapy exceed 90% in modern trials incorporating advanced imaging (4, 5). This comes at a modest cost, with rates of toxicity that are low, specifically grade 3 or higher rates of less than 5%, and in some cases less than 2%. Historically gastrointestinal (GI) toxicity predominated, however recently urinary and sexual side effects have become the predominate concern for patients (5–7). While RT is already highly effective, and minimally invasive, there are opportunities for improvement. Advances in technology are rapidly enabling this, and it’s imperative that radiation oncologists consider these advances and how they may optimally be applied to improve outcomes for men with localized prostate cancer. Radiation is dramatically and rapidly changing. Radiation oncologists are tasked with understanding novel advances in RT technology, and specifically how these advances could benefit their patients.

The current standard by which the vast majority of prostate cancer patients are treated uses computed tomography (CT) to guide treatment. Often a magnetic resonance image (MRI) is registered to the CT to further delineate treatment volumes. A CT is then acquired on the treatment machine. In other words, an “on board” cone beam CT scan (CBCT) is acquired to align the patient, often daily, and subsequently evaluate for rectal position, bladder size, and location of the prostate gland and target volumes intended for treatment with RT. This represents a current standard of care applied to thousands of men globally with a high degree of success. Outcomes using CBCT based image guidance are truly excellent. Due to significant interfraction motion, as well as intrafraction motion, fiducial markers are often placed to help enhance the accuracy and precision of treatment, allowing smaller volumes of normal tissue to be irradiated. Fiducial placement is an invasive procedure, albeit a minor one, that carries risk of infection and bleeding and requires logistical support. There is also concern with this strategy that the invasive use of fiducials could rarely cause toxicity (8).

Magnetic Resonance Image (MRI) guidance is an emerging technology that enables routine access to adaptive RT. There are currently two available commercial devices, one by ViewRay (Oakwood Village, Ohio) (9) and a second by Elekta (Elekta AB, Stockholm, Sweden) (10). Key differences have been the subject of prior reivews (11). Broadly speaking across both devices, MRI guidance differs from simply MR registration, which involves aligning an MRI with a CT to facilitate soft tissue delineation, often only at a single time point such as CT simulation. Using MRI guidance involves acquiring an MRI daily before each treatment with RT to precisely guide, or align, the location of high RT dose. The process of MRI guidance also greatly facilitates adaptive RT which involves changing the dose of radiation to account for subtle changes in normal anatomy, often on a daily basis. The concept of adaptive RT has been recently addressed by multiple review articles (11, 12). In brief, this involves daily target volume and surrounding organ at risk contouring and plan recalculation to account for differences in normal organ and tumor position. Routine adaption represents a particularly promising future area for development in RT. This can be done with both CT or MR based solutions, yet MR imaging has superior soft tissue visualization, and CT presents challenges in visualizing local normal structures. However, adaptive radiotherapy is resource intensive with regards to cost and time, and may not be appropriate for all patients. Secondary to these limitations, MRI-guided RT is not universally available. MRI-guided RT remains a technology that is predominantly limited to high volume academic centers, yet is gaining traction within community centers. While these limitations also apply to CT-based methods of adaptation, MR guidance may require additional medical physics support and MR safety precautions. Precisely how this technology can improve outcomes for men with localized prostate cancer beyond highly effective CT based RT treatment strategy remains unclear. We sought to collect a group of radiation oncologists and medical physicists with the objective of organizing consensus around the optimal prospective testing of this technology for patients with localized prostate cancer. This will focus on current definitive prostate treatment trials, areas for improvement not enabled by CT, and future trial design considerations.

## Methods

To summarize the current clinical trial landscape, a literature search was performed for trials focused on MR guided RT for localized prostate cancer. Trials that used CT based treatment delivery strategies that incorporated registered diagnostic MRIs or MR simulations only (without MR guided treatment machines) were excluded by review of abstracts. Current data was abstracted from both PubMed, Google Scholar, and Clinicaltrials.gov. In addition, the Medical College of Wisconsin Libraries (MCW) conducted an Ovid Medline search including the following search terms: Magnetic Resonance Imaging, image guided radiation therapy* or image-guided radiation therapy* or image-guided radiotherapy* or radiotherapy target organ alignment were included in the Ovid search criteria. This was further limited to published prospective clinical trials in a sub search.

With the goal of organizing ideas, a central table was collected to formulate where precisely adaptive MR guided RT delivery strategies could improve the treatment of localized prostate cancer. The goal of this effort was to focus specifically on the use of MRI-guided RT, and not MR registration. Collaborative input on potential trial designs, and opportunities for improvement, were collected.

The PRISMA checklist was referenced when conducting the search for this effort, however this was intended to be a narrative review, consequently all criteria specified in PRISMA were not used. A search of clinicaltrials.gov was conducted for ongoing trials. In-general these search terms included (radiotherapy OR radiation therapy OR radiation therapies OR Radiation treatment OR radiation treatments) AND (mri OR magnetic resonance) AND (guided OR guidance) | Prostate Cancer. This search was further restricted to trials that were either recruiting or active and not yet recruiting. Specifically there were several trial concepts that were “in development” however had not yet opened or been made publicly available on clinicaltrials.gov. These were excluded from this summary table.

## Results

There were a total of 742 published articles that returned after the initial search query; these were individually reviewed and select articles are summarized in **Table 1** with citations. With regard to results from clincaltrials.gov, a total of 123 trials resulted from the search. Again, many of these trials involved registering MRI to a reference planning CT. Selected trials are also summarized in **Table 1**.

**Table 1:** Currently published prospective trials to have evaluated mr guided rt in localized definitively treated prostate cancer.

Published	Inclusion criteria/Design	Primary endpoint	Outcomes
Kishan et al. (13, 14) NCT04384770 “Mirage”	*Inclusion:* - Planned SBRT for localized prostate cancer. *Design:* - Single center, randomized, phase 3, superiority trial. - Randomly assigned (1-1) to either CT-guidance VS. MRI-guidance SBRT - Interim analysis at 100 patients [presented (13)] - Planned sample size of 154 patients - Dose: 40 Gy in 5 fractions	- Acute grade 2 or higher GU toxicity - Device: ViewRay	- Prespecified efficacy analysis has been reported (13) - Acute grade ≥2 GU toxicity was significantly reduced in men receiving MRI-guided SBRT (incidence of 24 (47.1%) vs. 11 (22.4%), p = 0.01) - Acute grade ≥2 GI toxicity was significantly reduced in when using MRI-guided SBRT (incidence of 7 (13.7%) vs. 0 (0%), p = 0.01.)
Tetar et al. (15) NCT03961321	*Inclusion:* -Localized prostate cancer, T1-3b, no lymph node involvement, or distant metastases, prostate volume of 90 cc, baseline IPSS of < or = 19 *Design:* - Prospective single arm phase II trial - Single center - Dose: 36.25 Gy in 5 fractions	- Main study parameter: Early and early-delayed toxicity (CTCAE v. 4.0); (IPSS) and Qol C30 PR25 - Device: ViewRay	- Increase in the QLQ-PR25 urinary symptom score at the end of RT and at 6 wk of follow-up (+15.8 and +7.4, respectively) - The QLQ-PR25 bowel score was increased at all time points - Rectal Bleeding was uncommon, with a maximum reported rate of 1.1% at any time - At 12 mo, 2.2% of patients reported a relevant impact on daily activities due to bowel problems (QLQ-PR25 question 10)
Leeman et al. (16) NCT04115254	*Inclusion:* *-* Prostate cancer planned for 5 fraction MR guided RT *Design:* *-* Phase I trial - Sample size of 10, A sample size of n = 10 was chosen for feasibility, defined as enrolling subjects and delivering adaptive MRI-guided RT. Feasibility was defined as using MR guidance for each treatment fraction, and generating adaptive plans - Dose: 36.25 Gy in 5 fractions - Median Follow Up: 7.9 months	- Safety and feasibility of prostate adaptive MRI-guided RT - Device: ViewRay	- Median follow-up time was 7.9 months (range, 3.3-22.0) - Safety and feasibility of the phase 1 cohort met - No grade ≥3 toxicity events were observed - Five patients experienced a grade 2 GU toxicity event (22.7%), which all occurred within the first 3 months of SBRT - EPIC-26 bowel and urinary incontinence scores did not change significantly from baseline to end of MRI-guided RT, to 3-months post-SBRT - EPIC-26 urinary obstructive scores decreased by a mean of 9.4 points between baseline and end of SBRT (*P* = .03)
Pathmanathan et al. (17) NCT03658525 “PRISM”	*Inclusion:* - Adenocarcinoma prostate- grade group 3 or less (Gleason 4 + 3 = 7 orless). - Staging T2-T3a,N0M0 (MRI or DRE staging allowed) - PSA < 25 ng/ml - Maximum prostate volume 70cc - IPSS <12 at baseline - WHO performance status 0 or 1 *Design*: - Single center, single arm feasibility study - Dose: 60 Gy in 20 fractions - Planned sample size of 30 participants	- The proportion of patients in whom the imaging and treatment on the MR Linac (i.e. total time on the treatment couch) can be completed within 1 hour on 90% of fractions as assessed by the radiotherapy timing sheet - Device: Elekta Unity	- Proportion of patients and proportion of fractionations which require adaptive replanning due to anatomical changes- Time taken for adaptive replanning- Emergent acute GU and GI toxicity up to 12 weeks post completion of radiotherapy- Cumulative late toxicity at 2 years and 5 years- Patient reported outcomes IPSS, EPIC and EQ5D- Patient acceptability of treatment on the MR Linac- Biochemical progression free survival (PSA) at 2 years and 5 years- PSA nadir
**Accruing**	**Inclusion Criteria/Design/Total N**	**Primary Endpoint/Device**	**Secondary Endpoints**
NCT04595019 “HERMES” (18)	*Inclusion:* - Men aged ≥18 years - Prostate adenocarcinoma requiring radical radiotherapy - Gleason score 3 + 4 or 4 + 3 (Grade groups 2 or 3) - MRI stage T3a or less, PSA <25 ng/ml prior to starting ADT (Androgen deprivation therapy)concurrent androgen deprivation therapy (ADT) for at least 6 months, as per standard of care. *Design*: -Single center, non-comparative randomized phase 2 trial Randomly assigned (1:1) to either MRI-guided radiotherapy, 24 Gy in 2 fractions (boost to 27 Gy to tumour GTV) over 8 days VS. MRI-guided radiotherapy, 36.25 Gray (Gy) in 5 fractions (boost 40 Gy to prostate CTV) over 10 days. - Planned sample size of 46 participants	-CTCAE Grade 2+ genitourinary (GU) toxicity from the start of radiotherapy up to 12 weeks post-treatment Device: Elekta Unity	- Quality of life patient-reported outcomes: IPSS, EPIC-26, EQ-5D (EuroQol-5D) and IIEF-5 (International Index of Erectile Function) - Physician-reported CTCAE Genitourinary (GU) and Gastrointestinal (GI) toxicity - PSA (Prostate Specific Antigen) control and biochemical failure/progression - Imaging response using mpMRIs at baseline, 2 weeks post RT, 12 weeks post RT - Blood for immune profiling at baseline, immediately post-RT, 3, 6, and 12 months
NCT05373316 “AFFIRM”	*Inclusion*: - MRI stage iT3b - N0M0 on PSMA-PET - Intraprostatic lesion visible on MRI - IPSS <15 - PSA ≤30 - Prostate volume ≤100cc *Design:* - Single arm, phase II multicenter study: - Dose: 5x7Gy + focal boost up to 50Gy - Planned sample size 95 patients	Acute GU and GI toxicity Device: Elekta Unity and Viewray MRIdian	- Quality of life patient-reported outcomes: QLQ30 and PR25 - Physician-reported CTCAE Genitourinary (GU) and Gastrointestinal (GI) late toxicity - PSA (Prostate Specific Antigen) biochemical failure - Distant metastatic failure - Overall and prostate cancer specific survival
NCT04075305 “MOMENTUM”	*Inclusion:* -Patients with brain, lung, esophageal, breast, head and neck, pancreatic, gynecologic, rectal, prostate, bladder, oligometastatic, or liver cancer undergoing radiation therapy *Design:* - Prospective registry study - Currently over 700 prostate patients accrued, over 2000 total patients.	-Not formally powered Device: Elekta Unity	-Extensive, and tumor site dependent, previously published (19, 20).
NCT04984343 “FORT”	*Inclusion:* - Men aged >=18 with histologically confirmed low or intermediate risk prostate cancer per NCCN guidelines. - ECOG 0 - 1 - IPSS < 18 - Ability to receive MRI-guided radiotherapy. - Ability to complete the Expanded Prostate Cancer Index Composite (EPIC) questionnaire. - Patients with a prior or concurrent disease whose natural history or treatment does not have the potential to interfere with the safety or efficacy assessment of the investigational regimen are eligible for this trial. *Design:* - Randomized Phase II Trial of Five (37.5 Gy in 5 fractions) or Two (25 Gy in 2 fractions) MRI-Guided Adaptive Radiotherapy Treatments for Prostate Cancer with optional integrated boost - Plan to accrue 136 participants, randomized 1:1 to either 2 or 5 fractions	Primary endpoint: -Change in the number of patient-reported GI symptoms using the Expanded Prostate Cancer Index Composite (EPIC)[Time Frame: Baseline, 24 months] Device: ViewRay	- Change in GI symptoms at specific intervals, EPIC at baseline, 3, 6, 12, and 60 months. - Change in GU specific symptoms at time intervals, EPIC at baseline, 3, 6, 12, 60 months. - Change in sexual symptoms, EPIC baseline, 3, 6, 12, 60 months. - Time to progression - Overall survival - Prostate cancer specific survival
NCT04402151 “PSMART”	*Inclusion:* - Male aged 21 years or older. - Pathologic confirmation of high-risk adenocarcinoma of the prostate gland as follows: a. Gleason 8-10 or tertiary component 5 disease and/or b. PSA of 20 ng/ml or greater and/or c. Tumor stage of T2c or greater; OR Unfavorable intermediate risk (Gleason 4 + 3 = 7, >50% of cores involved, or 2 or more intermediate risk factors which include Gleason 7 disease, PSA 10-20, or T2b disease) - Participants must agree to use an acceptable form of birth control and utilize condoms for a period of seven days after each PSMA injection, if engaged in sexual activity. - No evidence of metastatic disease, including pelvic lymph nodes. *Design:* - PSMA PET/MR Guided Stereotactic Body Radiation Therapy With Simultaneous Integrated Boost (SBRT-SIB) for High-Intermediate and High Risk Prostate Cancer - Planned accrual of 50 patients.	Primary endpoint: -2 year recurrence free survival Device: ViewRay	- Performance of PSMA PET/MR to MR alone at staging prostate cancer - Performance of PSMA PET/MR to MR alone for identification of dominant intraprostatic nodules during radiation planning - Compare imaging biomarkers of interest on MR and PSMA PET/MR as predictors of treatment response, versus biopsy of treatment response and PSA - Compare imaging biomarkers of interest on MR and PSMA PET/MR as predictors of treatment response, versus biopsy of treatment response and PSA
NCT04845503 “SMILE”	*Inclusion:* - Histologically confirmed prostate carcinoma with tissue classification according to Gleason score and PSA - Low- or intermediate-risk carcinoma according to d’Amico criteria or early high-risk Carcinoma (cT3a and/or GS ≤ 8 and/or PSA ≤ 20ng/ml) - IPSS (International Prostate Symptom Score) max of 12 - Prostate volume <80cm³ - Karnofsky index ≥ 70% - Age ≥ 18 years - Patient information provided and written consent - Ability of the patient to give consent *Design*: - Prospective, non-randomized, multicenter, Phase II testing 37.5 Gy in 5 fractions - Planned accrual of 68 patients.	-Toxicity or Discontinuation of Therapy [Time Frame: Within 1 Year] One of the following events are counted as an Event: Any urogenital or gastrointestinal grade ≥ 2 toxicity within one year after the start of RT (according to NCI CTCAE Version 5.0) Discontinuation of therapy, with a connection to the study treatment Device: ViewRay	- Mortality - Biochemical Progression Free Survival - Hormone Therapy Free Survival - Overall Survival - Quality of life using the EORTC QLQ-C30 - Quality of life using the EORTC QLQ-PR25 - Symptoms and Toxicity (NCI CTCAE)
NCT04896801 “PROSEVEN”	*Inclusion:* - Age > 18 y - Histologically confirmed prostate adenocarcinoma - Low risk: cT1c-T2a, Gleason score 6, PSA < 10ng/mL - Favorable intermediate risk: 1 intermediate risk factor, Gleason 3 + 4 or less, < 50% positive biopsy cores) - Unfavorable intermediate risk: > 1 intermediate risk factor, Gleason 4 + 3, > 50% positive biopsy cores) - Limited high risk: cT3a with PSA < 40ng/mL or cT2a-c with a Gleason score > 7 and/or a PSA > 20ng/mL but < 40ng/mL - World Health Organization performance score 0-2 *Design:* - Prospective, non-randomized, Patients will be treated in 5 daily fractions within a short overall treatment time (OTT) of 7 days with a boost to the dominant lesion, PTV will receive 36 Gy in 5 fractions, the GTV will receive up to 42 Gy in 5 fractions - Planned accrual of 120 patients	-Clinician reported grade 2 or more acute gastrointestinal (GI) and genitourinary (GU) toxicity, assessed using CTCAE v 5.0 and RTOG, measured up to 3 months after the first treatment fraction.	- Late toxicity, CTCAE v 5.0 - Late toxicity according to RTOG criteria. - EORTC QLQ C30 quality of life. - EPIC-26 quality of life - IPSS quality of life - Freedom from biochemical failure - Disease-free survival - Overall Survival
NCT04861194 “ERECT”	*Inclusion:* - Age ≥18 years - Histologically proven adenocarcinoma of the prostate - Low-risk or intermediate-risk prostate cancer according to NCCN risk categories (low risk: T1c-T2a, Gleason score ≤6, and PSA <10 µg/L; intermediate risk: T2b-T2c or Gleason score 7 or PSA 10-20 µg/L) - Patients with pT1a/b tumor diagnosis after transurethral resection of the prostate (TURP) - Domain score of 17-25 on the International Index of Erectile Function-5 (IIEF-5) questionnaire - Karnofsky score of 70-100 - Written informed consent	-Erectile function score of ≤11 on the International Index of Erectile Function (IIEF) -5 questionnaire (0=worst; 25=best)	- Relapse free survival - Patient reported quality of life - Acute and late gastrointestinal and genitourinary toxicity
NCT04997018	*Inclusion:* - Age ≥ 18 - Karnofsky Performance Status (KPS) ≥ 80 - Prostate size ≤ 80 cc - Presence of a T2-visible prostatic lesion with maximum dimension of ≥ 0.5 cm and no more than one additional disease focus - MRI findings: Lesion may contact the capsular edge, possible extracapsular extension (ECE) permitted - International Prostate Symptom Score ≤ 15	-To demonstrate efficacy of dose escalation to the DIL, the investigators aim to reduce the positive post-treatment biopsy rates at 24 months for intermediate risk disease from 20% to 10%	

IPSS, International Prostate Symptom Score; EPIC-26, Expanded Prostate Index Composite-26.

Potential strategies for improvement with MR guidance in localized prostate cancer were reviewed and tabulated accounting for input amongst all authors. These are presented in **Table 2** for conceptual consideration and future trial design consideration. Moreover, this table also includes hypotheses that could be addressed with the use of MR guidance specifically over CT based image guidance. Finally, novel trial design strategies, with possible endpoints were suggested in **Table 3**.

**Table 2:** a: Opportunities for Improvement in localized prostate cancer when considering adaptive MR guided RT trial designs.

Challenge with CT based treatment	Potential for MR guided or Adaptive RT to improve outcomes, potential hypothesis to be addressed by MR Guidance	Technological innovation enabling hypothesis testing	Current References/Baseline using CT based RT
Sexual Dysfunction is common following CT based RT	- *Challenge*: Visualization of structures associated with sexual function is very difficult with CT based image guidance. - *Hypothesis:* MR Guided RT enables improved visualization and sparing of organs associated with male sexual function (neurovascular bundle, corpus cavernosum and spongiosum, internal pudendal artery) - *Potential Endpoints:* Patient reported sexual function	-MRI enables visualization, and potentially improved sparing, of nervous and vascular structures associated with sexual function.	(3, 6, 21–23)
There exists unpredictability of intra-fraction motion (aka beam on motion)	- *Challenge*: Considerable prostate and normal organ movement takes place during the “beam on” time with CT guided RT, this is difficult to visualize with CT based image guidance - *Hypothesis:* The use of MR guidance will enable normal organ visualization during RT delivery and therefore enable treatment interruption or adjustment if large shifts are appreciated. - *Potential Endpoints:* Number of events during treatment felt to necessitate termination of treatment	-MRI during beam on enables visualization of normal prostate tissue, should movement occur this can be accounted for, allowing margin reduction -However, benefit of this technique remains uncertain (24)	(25, 26)
Precise dosimetric data over a full treatment course is variable and uncertain	- *Challenge*: In non adaptive RT, there are frequent movements of regional OARs during a course of treatment for prostate cancer; the actual delivered DVH (versus the initial planned DVH) to regional OARs is uncertain, when considering focal dose escalation this is especially important. - *Hypothesis:* MR Guided RT enables improved visualization and DVH certainty by allowing daily plan recalculation as compared with CT guided RT, which has limited visualization - *Potential Endpoints:* Late GI and GU toxicity events directly comparing CT and MR based treatment strategies, OR patient reported QOL (perhaps as a co-primary endpoint)	-MRI during treatment shows prostate swelling, rotation, or rectal and/or bladder displacement not appreciated on CT	(27–29)
Selective focal RT dose escalation, either focal boosting or brachytherapy	- *Challenge*: There are currently no validated strategies to understand an individual patient’s response to RT, during the course of RT, and potentially optimally select patients for more aggressive RT treatment strategies (such as brachytherapy boosting). It is also challenging using CT to focally boost portions of the prostate, and de-escalate others based on response. - The routine use of brachytherapy boosting remains controversial and is associated with a higher rate of side effects - DWI may hold promise in this regard, but additional investigation is needed, especially when using MRI - *Hypothesis:* Specific DWI changes during a course of RT predict for future local failure events, allowing for adjuvant therapy changes (eg. introduction of adjuvant ADT, or brachy boosting) - *Potential Endpoints:* Specific magnitudes of ADC change correlated with biochemical failure and prostate only recurrence (phase II endpoint)	-Theoretically, functional MR imaging, potentially diffusion weighted MRI daily, or other novel types of MRI, may be associated with response to radiation therapy (30, 31), however this has yet to be proven	(32–34)
Contouring the prostate and rectum and adapting is time consuming	- *Challenge:* Contouring of regional anatomy is time consuming and subjective. - *Hypothesis:* AI assisted contouring may improve speed and accuracy over human only contouring. - *Potential Endpoints:* Adaptive time required along with contour approval when comparing AI assisted to human contouring.	-MR based AI solutions may improve contouring speed and accuracy to a level that is not currently available using CT based AI solutions.	
The Presence of a PTV margin invariably involves treatment of normal organs and potentially increases toxicity	- *Challenge:* The historic notion of a PTV limits radiation oncologists. - *Hypothesis:* The presence of real time MR imaging could eliminate the need for a PTV entirely without compromising oncologic outcomes.	-Continuous MR acquisition during beam on may enable complete elimination of the PTV	

DVH, Dose Volume Histogram; RT, Radiation Therapy; ADC, apparent diffusion coefficient; OAR’s, Organs at risk; AI, Artificial Intelligence; PTV, Planning target volume.

**Table 3:** Potential trial designs for MR guided RT based interventional trials compared with CT.

Trial type	Rationale for use in MR guided RT studies	Reference
Early phase tumor basket/platform trials	- Pan-tumor studies may allow more rapid evaluation of technical/feasibility endpoints prior to disease-site specific clinical evaluation	(35)
Bayesian/model-based dose-finding designs	- Efficient assessment of dose escalation - Allow incorporation of time to event to incorporate late toxicity events accrued at point of dose escalation decision	(36)
Phase I/II	- Enables first dose intensification/dose finding followed by efficacy - Ideal for tumors that can be visualized more clearly with MR guidance in which dose escalation may be more helpful: phase I portion - Subsequent and seamless efficacy assessment in the form of cancer specific outcomes with the phase II portion	(37, 38)
Randomized Selection Design, Phase II “Pick the winner”	- Ideal in the setting of unknown magnitudes of benefit or absence hypothesized magnitudes of benefit at the outset - Useful in comparing optimal strategies to take forward for the Phase III setting - Helpful in informing the design of future phase III trials - Could be used to compare two approaches (example MR based sexual organ at risk sparing as compared with CT based) subsequent winning arm could advanced into phase III study	(39)
Trials Within Cohorts “TWICS” Design	- Utilize existing large observational cohorts, such as MOMENTUM (NCT04075305) - Repeated measurements of outcomes for the whole cohort of actively treated patients can be made as a benchmark comparison - Patients can be identified within the cohort and randomly assigned to interventions (with informed consent), outcomes can be compared to a contemporaneously enrolled cohort within the observational study.	(40)
Phase III randomized controlled parallel group trial	- Gold standard level 1 evidence - Patient focused endpoints required to drive practice change - Potential Randomization to CT guided vs MRgRT demonstrated as feasible	
Multi-stage, Multi-arm trials	- If intermediate endpoint available to support stop/go decisions (multi-stage) could be helpful in determining potentially useful treatment strategies (multi-arm) to pursue into phase III	(41)
Bayesian Basket trials	- Incorporate sharing or borrowing of information from different tumor baskets if radiobiology supports “similar” effects.	(42)

## Discussion

The ability to use MRI guidance has considerable promise for patients with localized prostate cancer (12, 43). At the present time these advantages are mostly theoretical, however data is emerging that is showing exciting improvements associated with the use of MRI-guided RT (13). A concerted focus on how to optimally demonstrate the advantages of MR guidance in localized prostate cancer is needed. There is an imperative to test this technology against current established standards. This imperative is critical for two overarching reasons. First, the current standard of care when using CT based RT is resulting in truly excellent outcomes with relatively low rates of toxicity. One such example is the recently published Prostate-Only Versus Whole-Pelvic Radiation Therapy in High-Risk and Very High-Risk Prostate Cancer (POP-RT) trial (4). In this study, the five year biochemical failure free survival was an impressive 95% when using PSMA PET combined with prostate and whole pelvic RT. This impressive rate of control was achieved with very acceptable toxicity rates (4). Carefully iterating on currently established standards of care, with a focus of how RT can be refined further, is essential. A second important imperative to consider is that the use of MR guidance is more logistically complex and potentially time consuming than CT based treatment. However, this could be considerably impacted by artificial intelligence (AI) based contouring solutions and the potential use of ultra-hypofractionation schedules. Interestingly, despite a commonly exchanged narrative, the use of MRI-guided RT technology does not appear to add considerable cost when accounting for potential differences in toxicity and fractionation schedules (44). Despite this, adopting this technology does not come without the very real potential for costs to both patients and healthcare systems. For both of these reasons, the magnitudes of improvement with MR based RT and adaptive therapy in localized prostate cancer are essential to study and quantify.

An important consideration in this discussion is that evaluating novel radiation technological innovations is difficult. Road maps and methodologies for introducing new RT techniques have been proposed (37). But conducting randomized trials comparing novel RT based technologies to historically established standards remains relatively rare. There are a few reasons for this. One is an absence of clearly tangible incentives for device manufactures to sponsor and conduct this type of research. There is minimal (to no) regulatory imperative from the Food and Drug Administration (FDA) for companies to conduct randomized trials robustly proving the added value of novel technologies. In addition, there are substantial costs associated with conducting a randomized trial, which often serves as a disincentive for device manufacturers to sponsor. The other challenge is that advantages may seem overwhelmingly apparent to some radiation oncologists who evaluate this novel technology. In fact, one may easily ponder the question: “if a device enables more clear visualization of a target, does such a device really require comparative randomized data before it can be adopted, particularly if it can be purchased and used without randomized evidence?” This is difficult to address in the real world setting of RT delivery. It is also important to consider that there is a common perception amongst radiation oncologists that many of these technologies are, at worst, equivalent to current treatment strategies using CT. Therefore, taking time to prove the magnitude of this advantage, as opposed to just adopting the technology (that has seemingly apparent advantages) presents a distinct obstacle that our field faces with exceeding frequency. Despite these barriers, there are some examples of radiation device manufacturers funding (but not sponsoring) novel interventional studies testing radiation treatment strategies in prostate cancer (45). As radiation oncologists we are often reminded of the age old adage that just because we can, does not mean we should. Its something that should be very carefully reflected on by all radiation oncologists.

The concept of when a randomized trial is needed has been recently debated (46). Indeed, precisely when a randomized trial is necessary is not entirely clear. There does not seem to be robust consensus on this issue. One proposal has been that a trial may be needed when an intervention is thought to be beneficial by physicians, but offers only modest benefit with the potential for harm (46). Another indication would be when a standard of care is debated, or poorly understood, early or mid-phase randomized trials provide an important strategy to identify an optimal approach. MR guided radiation certainly may fall into this category, and this could potentially be the case in localized prostate cancer where current CT based RT interventions offer remarkable success. We have proposed several conceptual areas in which CT based RT for localized prostate cancer could be uniquely improved by MR guidance. These range from sexual function improvements, enabled by better visualization of the structures enabling male sexual function to biologically adapted therapy based on changes in DWI. Each of these conceptual areas could uniquely leverage MR guided technology.

The current landscape of randomized trials in prostate cancer testing the benefits of MR guidance is modest. Extremely important trials, such as the MIRAGE trial (NCT04384770), have been pioneered recently and may set a standard moving forward making future randomization to CT based treatment difficult in some centers. More questions using MR guidance could be asked, perhaps with larger multi-center trials. MIRAGE has clearly and critically demonstrated that randomization of this population is feasible, but its results may make future randomization between MR and CT challenging (13). Moreover, such a trial provides strong data that acute toxicity can indeed be improved with the use of MR guidance over CT based image guidance. This is an important step toward future MR guided trials that could be larger, multi-center, randomized trial designs testing cost effectiveness and even further improve patient benefit. The US National Cancer Institute (NCI), Elekta MR Linac Consoritum, and ViewRay Consortium cooperative groups could play a central role in expanding and refining this portfolio. Industry sponsored consortium registries (such as MOMENTUM; NCT04075305) offer another opportunity for potentially conducting nested trials examining novel approaches within the spectrum of MR guidance. Trials could potentially test the ability of MR guidance to further improve endpoints including: late toxicity, sexual toxicity, or even further reduce the existing small magnitudes of acute toxicity. Such a trial should ideally be device agnostic and include enough patients to enable robust estimates of the magnitude of effect sizes. Such a proposal is in development and planned for submission in the coming year. Finally, there is an important need to both test and standardize dose schedules along with constraints of applied RT doses when possible.

## Conclusions

MRI guidance is rapidly gaining popularity, with over 150 devices installed from various vendors. The technology holds considerable promise for continuing to refine and improve outcomes in patients with localized prostate cancer. The specialty, and academic radiation oncologists, must remain vigilantly focused on proving this technologies value and throughput safely. While radiation oncologists may be enthusiastic to start using this technology on patients, we must simultaneously test novel hypothesis as to how this technological advance can improve their outcomes. The future is promising for the continued intersection of technological imaging advances and precision RT delivery.

## Author contributions

All authors listed have made a substantial, direct, and intellectual contribution to the work and approved it for publication.

## Conflict of interest

WH, EP, and AT receives research and travel support from Elekta AB, Stockholm Sweden. HN: ViewRay Ad board research funds BSci ad board and research funds Lantheus research funds Veracyte research funds. AK: ViewRay Research funding, honoraria, consulting Varian Honoraria Boston Scientific Advisory Board Janssen Research FundingPointBiopharma Research funding. EH: Prof. Hall reports grants from Accuray Inc., grants from Varian Medical Systems Inc., outside the submitted work; EH acknowledges support from a Cancer Research UK Network Accelerator Award Grant (A21993) to the ART-NET consortium; and from the NIHR Biomedical Research Centre at The Royal Marsden NHS Foundation Trust and ICR (London, UK). DV reports speaker fees from Elekta. AT acknowledges support from Cancer Research UK (C33589/A28284 and C7224/A28724) and the National Institute for Health Research (NIHR) Cancer Research Network. This project represents independent research supported by the National Institute for Health research (NIHR) Biomedical Research Centre at The Royal Marsden NHS Foundation Trust and the Institute of Cancer Research, London. The views expressed are those of the authors and not necessarily those of the NIHR or the Department of Health and Social Care.

The remaining authors declare that the research was conducted in the absence of any commercial or financial relationships that could be constructed as a potential conflict of interest.

## Publisher’s note

All claims expressed in this article are solely those of the authors and do not necessarily represent those of their affiliated organizations, or those of the publisher, the editors and the reviewers. Any product that may be evaluated in this article, or claim that may be made by its manufacturer, is not guaranteed or endorsed by the publisher.
